# Folate Metabolism Gene 5,10-Methylenetetrahydrofolate Reductase (*MTHFR*) Is Associated with ADHD in Myelomeningocele Patients

**DOI:** 10.1371/journal.pone.0051330

**Published:** 2012-12-05

**Authors:** Catherine J. Spellicy, Hope Northrup, Jack M. Fletcher, Paul T. Cirino, Maureen Dennis, Alanna C. Morrison, Carla A. Martinez, Kit Sing Au

**Affiliations:** 1 Department of Pediatrics, University of Texas Medical School at Houston, The University of Texas Health Science Center at Houston, Houston, Texas, United States of America; 2 Shriners Hospital for Children, Houston, Texas, United States of America; 3 Department of Psychology, University of Houston, Houston, Texas, United States of America; 4 Department of Psychology and Texas Institute for Measurement, Evaluation, and Statistics (TIMES), University of Houston, Houston, Texas, United States of America; 5 Department of Surgery, University of Toronto, and Program in Neurosciences and Mental Health, The Hospital for Sick Children, Toronto, Canada; 6 Human Genetics Center, Division of Epidemiology, Human Genetics and Environmental Science, School of Public Health, University of Texas Health Sciences Center at Houston, Houston, Texas, United States of America; 7 Department of Obstetrics and Gynecology, Texas Tech University Health Science Center, El Paso, Texas, United States of America; Kunming Institute of Zoology, Chinese Academy of Sciences, China

## Abstract

The objective of this study was to examine the relation between the 5, 10-methylenetetrahydrofolate reductase (*MTHFR*) gene and behaviors related to attention- deficit/hyperactivity disorder (ADHD) in individuals with myelomeningocele. The rationale for the study was twofold: folate metabolizing genes, (e.g. *MTHFR*), are important not only in the etiology of neural tube defects but are also critical to cognitive function; and individuals with myelomeningocele have an elevated incidence of ADHD. Here, we tested 478 individuals with myelomeningocele for attention-deficit hyperactivity disorder behavior using the Swanson Nolan Achenbach Pelham-IV ADHD rating scale. Myelomeningocele participants in this group for whom DNAs were available were genotyped for seven single nucleotide polymorphisms (SNPs) in the *MTHFR* gene. The SNPs were evaluated for an association with manifestation of the ADHD phenotype in children with myelomeningocele. The data show that 28.7% of myelomeningocele participants exhibit rating scale elevations consistent with ADHD; of these 70.1% had scores consistent with the predominantly inattentive subtype. In addition, we also show a positive association between the SNP rs4846049 in the 3′-untranslated region of the *MTHFR* gene and the attention-deficit hyperactivity disorder phenotype in myelomeningocele participants. These results lend further support to the finding that behavior related to ADHD is more prevalent in patients with myelomeningocele than in the general population. These data also indicate the potential importance of the *MTHFR* gene in the etiology of the ADHD phenotype.

## Introduction

Spina bifida (SB) is the most common disabling birth defect in North America, with a frequency of ∼3–4 in every 10,000 live births [1], [2], [3], [4]. The most common, and severe, form is myelomeningocele (MM), accounting for over 90% of all SB cases. MM is a spinal lesion characterized by an opening in the vertebral column through which the meninges and neural tissues protrude. Clinical consequences of the spinal lesion include difficulties with ambulation, sensation, incontinence, and learning problems [5], [6].

Attention deficits in MM individuals have been identified via attention deficit hyperactivity disorder (ADHD) ratings. Children with MM are more likely than controls to show clinically significant elevations on ratings of ADHD (31% in MM vs. 7% in the general population) [7]. Notably, 74.5% of MM individuals with significant elevations on ratings of ADHD were consistent with the predominantly inattentive subtype of ADHD [7]. An independent study by Ammerman *et al.* showed similar results [8].

Folic acid is a B vitamin important in relation to MM, as well. Studies show that folic acid supplementation decreases both the occurrence and recurrence of neural tube defects by 72% in seven countries [9]. Data such as this led to public health recommendations to supplement with folate periconceptionally, and in 1998 to fortify all grain products in the United States with folic acid [10].

**Figure 1:**
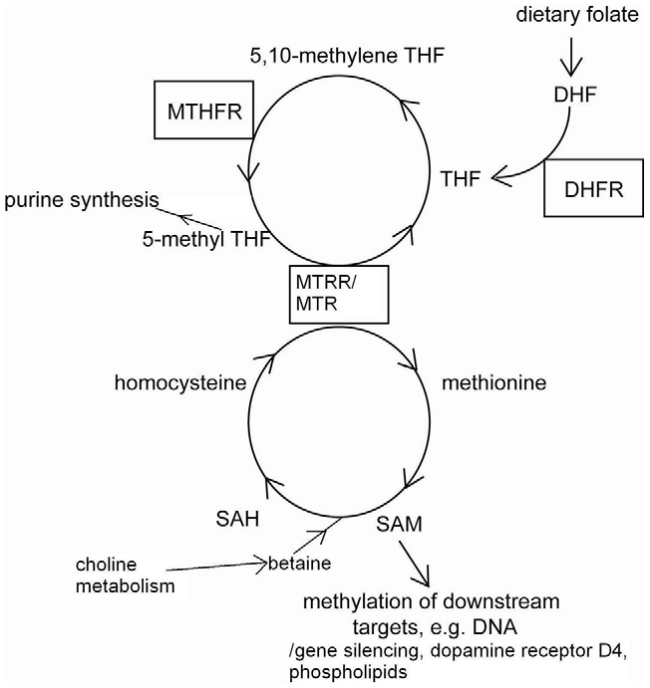
Simplified schematic of the folic acid metabolic cycle. Folate receptors transport dietary folate into cells and the folate is converted into dihydrogolate (DHF) then tetrahydrofolate (THF) by dihydrofolate reductase (DHFR). In the folate metabolic cycle, THF is converted to 5,10-methyleneTHF, a substrate of 5,10-methyleneTHF reductase (MTHFR), then to 5-methylTHF. 5-methylTHF can be recycled by methionine synthase/methionine synthase reductase (MTR/MTRR) to THF and methionine. Alternatively, 5-methylTHF can be use to synthesize purine. The Methionine can be used in the methionine cycle to produce S-Adenosyl-methionine (SAM), S-adenosyl-homocysteine (SAH) and homocysteine. Conversion of SAM to SAH requires betaine, a product of choline metabolism. SAM is a major cellular methylation agent for DNA, RNA, protein, and phospholipids.

Folic acid is critical to cellular processes including nucleotide synthesis and methylation. The enzyme, 5, 10-methylenetetrahydrofolate reductase (MTHFR) functions in the pathway that converts folate into metabolites that may be used for cellular processes including methylation of gene promoter enhancers and proteins, RNA, DNA, amino acid and phospholipids synthesis (see **Figure 1**). For example, dopamine-stimulated phospholipid methylation (PLM) is suggested to be an important mechanism to modulate firing of neurons and impaired methylation activity can contribute to attention disorders [11]. Neuroblastoma cells (SH-SY5Y) treated with 5-formylTHF caused an increased dopamine receptor D4 (DRD4) methylation by protein methyltransferase and S-adenosyl-methionine resulting in a dose-dependent increased in both basal and dopamine-stimulated PLM [12]. *DRD4* is one of the most replicated genes identified to associate with ADHD [13].

Genetic variants in *MTHFR* have been associated with MM [14], [15], [16]. The two most common variants studied are C677T (rs1801133) and A1298C (rs1801131). The presence of either of these variants in a homozygous state increases the odds of being affected with SB to 1.5 to 2.4 times that of the general population, although the data for 1298C is conflicting [17], [18], [19].

Several studies link folate/homocysteine levels with cognitive functions. For example, patients with folate deficiencies in the central nervous system exhibit cognitive deficits [20]. Morris *et al.* showed that, relative to controls, individuals with low folate had significantly reduced memory function [21]. Other studies show similar results; that is, cognitive functions improve with higher folate levels, and decrease with higher homocysteine levels. MTHFR is a key regulator of folate versus homocysteine levels [22], [23], [24], [25], [26].

In this study we tested whether genetic variation in the folate metabolism enzyme *MTHFR* was associated with manifestation of the ADHD behavioral phenotype in children with MM.

## Materials and Methods

### Participants

The participants in this study were an expanded population from that used in Burmeister *et al*. [7]. The sample genotyped for SNPs in the *MTHFR* gene consisted of 262 MM individuals. Fifty percent of these individuals were White and 50% were Hispanic. The sample consisted of 53% females and 47% males and median age was 12.4 years.

Parent rating scales for ADHD were also completed on additional patients for whom DNA was not available. These patients were used to compare frequency of positive ADHD test results in MM individuals versus positive ADHD tests in controls. For this comparison, the group sizes were as follows: the MM sample consisted of 478 patients and the control sample consisted of 196 control individuals not affected with MM. Ethnicity of individuals with MM and the control individuals without MM are listed in **Table 1**. Both samples consisted of approximately 50% males and 50% females with a median age of 13.06 years in the MM patients, and a median age of 12.73 years in the control group.

**Table 1:** Ethnicities of individuals in the study.

Americans	Individuals with MM			Individuals without MM		
	total	No ADHD	ADHD	total	No ADHD	ADHD
White	233	148	85	135	123	12
Hispanic	180	144	36	27	24	3
African	41	29	12	6	4	2
Asian	12	9	3	18	18	0
Other	12	9	3	10	10	0
**total**	478	339	139	196	179	17

These experiments were undertaken with the understanding and written consent of each subject abiding by the Code of Ethics of the World Medical Association. The study was approved by the University of Texas Health Science Center at Houston's Institutional Review Board under approval number HSC-MS-00-0001 and The Hospital for Sick Children Research Ethics Board approval number 1000006149.

### Assessment of ADHD Behavior

The parents of each participant completed the Swanson Nolan Achenbach Pelham-IV (SNAP-IV; www.adhd.net) rating scale to assess ADHD status. The SNAP-IV scale consists of 18 items, 9 representing behaviors associated with inattention and 9 with hyperactivity-impulsivity. It is designed to align with the criteria in the Diagnostic and Statistical Manual-IV (DSM-IV), widely accepted as a gold standard for diagnosis of ADHD. Parents rated their child's behavior on a four point scale, from zero (not at all) to three (very much), as previously described [7]. Participants who met clinically-defined cutoffs representing the upper 5% of the population on the inattention scale were designated “ADHD- Predominantly Inattentive Type”; those with elevations in the upper 5% only on the hyperactivity-impulsivity scale represented the “Hyperactive-Impulsive Type”. Elevation on both scales represented “ADHD Combined Type”. The SNAP-IV is a reliable parent-based assessment of ADHD behavior with good concordance to structured interviews [27], widely used to assess ADHD in clinical trials and in numerous researches studies in the US and Worldwide [28].

### Genotyping

**Figure 2:**
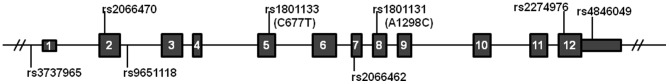
Genomic structure of the *MTHFR* gene and the location of the seven SNPs examined in this study. Shaded boxes represent the exons (1 to 12) of *MTHFR* gene and the line in between represent intron regions. Distances and locations are approximate.

SNP selection, genotyping, and statistical analysis were performed as previously described [29], [30]. Briefly, SNPs were selected within the reference *MTHFR* gene (NM_005957) from the University of California Santa Cruz (UCSC) Genome Browser dbSNP Build132 database as well as the HapMap3 public release #27. SNPs were prioritized for genotyping based on potential function (e.g. if the variant changes the amino acid), gene location, and a minor allele frequency greater than 0.05. A total of 7 SNPs in the *MTHFR* gene were selected (see **Figure 2** for approximate locations): rs3737965, rs2066470, rs9651118, rs1801133 (also known as 677C>T), rs1801131 (also known as 1298A>C, rs2274976, and rs4846049.

DNA was collected from MM patients for analysis. Genotyping was performed using the SNPlex platform on the ABI3730 genetic analyzer (Applied Biosystems Inc., Carlsbad, California, USA; ABI). Genotype calls were performed using the GeneMapper v4.0 software from ABI. For case-control analyses, MM participants with scores consistent with ADHD were defined as affected and were compared to MM participants without evidence of ADHD behavior.

### Statistical analysis

Genotype-phenotype association was examined by logistic regression using PLINK [31]. The sample was divided into ethnic strata (White and Hispanic) to avoid artifacts due to difference in genotype frequency between ethnicities. The critical level of alpha adopted for statistical significance was *p*≤0.05 and multiple testing effect was evaluated via permutation of disease status to generate empirical p-values. Linkage disequilibrium (LD) between two SNPs was evaluated using Haploview4.2 and significant LD is concluded with a correlation coefficient (r∧^2^) ≥0.8. Association of haplotypes of the six SNPs is examined using Haploview4.2 and the significance is evaluated by the 10,000 permutations.

## Results

### Behavioral testing

Results of ADHD assessment showed that of the 478 MM participants rated, 137 had clinically significant elevations on the SNAP-IV (28.7%). Of the MM participants with scores consistent with ADHD thresholds, the vast majority had elevations on the inattention scale associated with “ADHD Predominantly Inattentive-Type” (N = 96; 70.1%). Only 17 MM participants had elevations on the hyperactive-impulsive scale (12.4%) and 24 had elevations on both scales (21%). Of the 197 controls not affected by MM, 17 had significant elevations on any one or both of the SNAP-IV scales (8.6%). Of these 17 control individuals, 7 had elevations on inattention scale (38.9%), 3 had elevations on the hyperactive-impulsive scale (16.7%) and 7 had elevations on both scales (38.9%).

### Genotype/Phenotype association

Genotype-phenotype association analyses were performed using only the 478 MM affected individuals between a group with elevated score for ADHD and another group with score below the threshold. The numbers of White and Hispanic MM individuals genotyped for ADHD association analyses are listed in **Table 2**. Allele frequencies of *MTHFR* SNPs were evaluated for deviation from Hardy-Weinberg equilibrium (HWE) expectations (*p*≤0.05). All SNP genotypes were in HWE except rs9651118 in Hispanics (*p*<0.01), and this SNP was therefore eliminated from further analysis in that group (**Table 3**). Logistic regression models identified one of the seven SNPs in the *MTHFR* gene that was significantly associated with the ADHD phenotype (see **Table 4**). Specifically, the T allele of SNP rs4846049 in the 3′ untranslated region (3′-UTR) (see **Figure 2**) of the *MTHFR* gene is associated with risk for the ADHD phenotype with an empirical *p*-value of 0.00687 in the White MM participants tested. None of the tested SNPs showed association with ADHD in the Hispanic MM participants tested (**Table 5**). The rare allele frequency of rs4846049 is not significantly different between the White MM participants and the Hispanic MM participants; therefore, we performed association analyses of this SNP using data combing all the White and Hispanic MM participants. An association of the T allele of rs4846049 with ADHD in the combined group of all MM participants was observed with empirical p-value of 0.0474 (Odd ratio of 1.564; confidence interval of 1.014–1.475).

**Table 2:** White and Hispanic MM individuals genotyped.

Americans	rs1801131 & rs1801133			Other SNPs*		
	total	No ADHD	ADHD	total	No ADHD	ADHD
White	213	136	77	126	72	54
Hispanic	152	123	29	120	98	22

* – other SNPs include rs3737965, rs2066470, rs9651118, rs2274976, and rs4846049.

**Table 3:** Allele frequency of seven SNPs in the *MTHFR* locus of White and Hispanic MM individuals in the study.

				Whites			Hispanics		
dbSNP ID	Func.	A1	A2	N	Freq.	HWE	N	Freq.	HWE
rs3737965	promoter	C	T	126	0.94/0.06	NS	117	0.92/0.08	NS
rs2066470	p.P39P	C	T	123	0.90/0.10	NS	120	0.90/0.10	NS
rs9651118	intron	C	T	115	0.82/0.18	NS	109	0.72/0.28	<0.01
rs1801133	p.A222V	C	T	209	0.62/0.38	NS	145	0.49/0.51	NS
rs1801131	p.E429A	A	C	213	0.69/0.31	NS	152	0.86/0.14	NS
rs2274976	p.Q594R	G	A	117	0.96/0.04	NS	108	0.97/0.03	NS
rs4846049	3′-URT	G	T	120	0.78/0.22	NS	114	0.81/0.19	NS

Notes: rs – reference identification number of SNPs in the dbSNP database, Func. – functional significance of SNP, A1 – common allele of SNP, A2 – rare allele of SNP, N – number of subject successfully genotyped, Freq. – frequency of A1/2, HWE – Hardy Weinberg Equilibrium test result, NS – not significantly deviated from Hardy Weinberg Equilibrium (p>0.05). Additional samples were genotyped for rs1801133 and rs1801131 because these two SNPs have previously been examined with suggested association to ADHD [42].

**Table 4:** Case-Control Analyses of ADHD phenotypes in White subjects with MM.

dbSNP ID	N	Odds Ratio	95% CI	*p*-value	Empirical *p*-value
rs3737965	126	0.471	0.12–1.86	0.2830	0.3330
rs2066470	123	0.707	0.27–1.82	0.4720	0.5260
rs9651118	115	0.734	0.36–1.48	0.3890	0.4400
rs1801133	209	1.212	0.78–1.89	0.3960	0.5710
rs1801131	213	0.833	0.54–1.28	0.4030	0.8570
rs2274976	117	2.274	0.44–11.78	0.3280	0.4400
rs4846049	120	2.068	1.17–3.65	**0.0121**	**0.0069**

Note: N – number of subjects successfully genotyped. Cases are MM individuals testing positive for ADHD, controls are MM individuals testing negative for ADHD. Significant p-values ≤0.05 are indicated by bold lettering. Empirical p-value is the p-value obtained through random permutation of the experimental data to evaluate the effect of multiple testing.

**Table 5:** Case-Control Analyses of ADHD phenotypes in Hispanic subjects with MM.

dbSNP ID	N	Odds Ratio	95% CI	*p*-value	Empirical *p*-value
rs3737965	117	0.513	0.11–2.43	0.4000	0.3610
rs2066470	120	0.826	0.27–2.57	0.7410	0.5630
rs1801133	145	0.430	0.15–1.27	0.1260	0.1530
rs1801131	152	1.378	0.79–2.41	0.2600	0.2240
rs2274976	108	1.220	0.13–11.03	0.8600	0.4580
rs4846049	114	0.509	0.19–1.37	0.1820	0.1760

Note: N – number of subjects successfully genotyped. Cases are MM individuals testing positive for ADHD, controls are MM individuals testing negative for ADHD. Significant p-values are indicated by bold lettering. Empirical p-value is the p-value obtained through random permutation of the experimental data to evaluate the effect of multiple testing.

To test whether rs4846049 is in LD with the other five SNPs, we performed LD analyses using Haploview 4.2 among Whites and Hispanics with MM separately. The results showed r∧^2^ <0.8 for all tested SNPs (see **Table 6**).

**Table 6:** Linkage disequilibrium analysis of rs4846049 versus the other six SNPs tested.

SNP1	SNP2	SNP1 Chr1 Loc.	SNP2 Chr1 Loc.	HapMap CEU r∧^2^	White MM r∧^2^	Hispanic MM r∧^2^
rs4846049	rs3737965	11850365	11866451	0.10	0.09	0.11
rs4846049	rs2066470	11850365	11863057	0.22	0.21	0.29
rs4849049	rs1801133	11850365	11856378	0.19	0.15	0.11
rs4846049	rs1801131	11850365	11854476	0.93	0.63	0.70
rs4846049	rs2274976	11850365	11850927	0.10	0.10	0.14
rs4846049	rs4846049	11850365	11850365	1.00	1.00	1.00

Notes: Chr1 – Chromosome 1, Loc. – location in bases from p-arm of chromosome 1 with reference to human genome sequence GRCH37/hg19 assembly, r∧^2^ – correlation coefficient between SNP1 and SNP2, a value ≥0.8 suggests linkage disequilibrium.

We also perform haplotypes analyses of Whites subjects with MM on the six SNPs using Haploview 4.2 and observed significant between the rare allele T of rs4846049 with one of the major alleles of the other five tested SNPs (**Table 7**). In addition, a four SNPs haplotypes (CCGT) with the major alleles of rs3737965, rs2066470, rs2274976 and the rare allele T of rs4846049 also showed significant association. Haplotypes with other combinations of three, four five and six SNPs did not reach significant level by permutation (data not shown). Analyses of the haplotypes of Hispanics subjects with MM did not reach significant level (data not shown).

**Table 7:** Haplotype analyses of *MTHFR* SNPs and ADHD in White subjects with MM.

Haplotype	Total freq	Freq in ADHD	Freq in no ADHD	Chi Square	p-value	permutation p-value*
rs3737965, rs4846049						
CG	0.709	0.640	0.761	4.317	**0.038**	0.088
CT	0.242	0.323	0.180	6.645	**0.010**	**0.022**
TT	0.044	0.036	0.049	0.253	0.615	1.000
rs2066470, rs4846049						
CG	0.705	0.640	0.755	3.795	0.051	0.181
CT	0.193	0.268	0.136	6.736	**0.009**	**0.031**
TG	0.011	0.002	0.017	1.249	0.264	0.764
TT	0.090	0.089	0.092	0.006	0.940	1.000
rs1801133, rs4846049						
TG	0.371	0.325	0.405	1.649	0.199	0.462
CT	0.258	0.329	0.204	4.892	**0.027**	0.071
CG	0.344	0.313	0.367	0.794	0.373	0.742
TT	0.028	0.033	0.023	0.216	0.643	0.955
rs1801131, rs4846049						
AG	0.663	0.601	0.711	3.275	0.070	0.196
AT	0.032	0.060	0.011	4.708	**0.030**	0.098
CG	0.053	0.041	0.062	0.523	0.470	0.873
CT	0.252	0.298	0.216	2.151	0.143	0.412
rs2274976, rs4846049						
GG	0.713	0.637	0.770	5.017	**0.025**	0.090
GT	0.247	0.331	0.184	6.751	**0.009**	**0.043**
AT	0.040	0.032	0.046	0.297	0.586	0.981
rs3737965, rs2066470, rs2274976, rs4846049,						
CCGG	0.706	0.641	0.755	3.781	0.052	0.117
CCGT	0.188	0.255	0.136	5.594	**0.018**	**0.041**
CTGT	0.041	0.046	0.037	0.131	0.718	1.000
TTAT	0.035	0.023	0.044	0.790	0.374	0.907
TTGT	0.010	0.014	0.008	0.182	0.670	1.000

Notes: Haplotypes are generated by Haploview 4.2, freq – frequency of haplotypes, ADHD –Attention Deficit Hyperactivity Disorders.

* results of 10,000 permutations were performed using Haploview4.2 on. Significant p-values ≤0.05 are indicated by bold lettering.

## Discussion

Folic acid is a critical nutrient for gestational development as evidenced by the finding that neural tube defects were reduced by 19–55% after fortification of grain products with folic acid [32]. Additionally, folates have long been hypothesized to be important to central nervous system function and development. Lack of dietary nutrients such as folate and vitamin B has been associated with the development of neurodevelopmental disorders including attention deficit disorder/attention deficit hyperactivity disorder and autism [33]. Impaired methylation of dopamine receptor and membrane phospholipids can contribute to problem in firing of neurons and subsequently to attention disorders [11].

In addition, higher folate levels have been associated with slower cognitive decline in the elderly [26]. Elderly individuals who took daily folic acid supplements showed significantly improved global cognitive functioning [23]. In a non-elderly population of school-aged girls, it has been shown that taking iron and folic acid supplementation twice weekly improved cognitive skills [25]. Lastly, the Rotterdam study showed that cognition and psychomotor speed was positively correlated with higher plasma folate levels [22].

Children with MM have a pattern of preserved cognitive skills and cognitive-academic deficits [34], [35]. In MM populations, specifically, attention problems have been reported in a range of assessment contexts [36]. On cognitive tasks, children with MM have difficulty orienting to, and disengaging from external stimuli and they fail to inhibit attending to previously explored locations [37]. Children with MM make more sustained attention errors than controls [38].

The above studies suggest a complex relation among folate metabolism, neural development, and cognitive/behavioral function. The specific underpinnings of these relationships are not yet understood, but may be especially relevant for MM given the association of folate metabolism with the MM phenotype, as well as the increased incidence of attention problems and ADHD in MM patients.

Results of this study confirmed previous reports that the incidence of ADHD behavior is more than three times higher in MM populations (28.7%) than in the general population (approximately 8%) [39]. In addition, the proportion of inattentive behaviors in the MM population is almost twice that observed in the ADHD-affected control population (70.1% and 38.9% respectively). One of the seven SNPs tested in this study, rs4846049 in the 3′-UTR of the MTHFR gene, was significantly associated with ADHD behaviors in MM individuals. These findings provide a possible connection between variants of *MTHFR* gene, folate deficiency, MM and ADHD phenotypes and may help explain some of the variability in attention outcomes. More broadly, the data suggest avenues for future research involving the functional link between folic acid and specific behaviors. MM may represent a model disease for investigating the genetic etiologies and developing therapies for ADHD, at least for the inattentive subtypes potentially relating to folate/B12 deficiency.

Association studies of the *MTHFR* A1298C allele and ADHD are small, limited and inconclusive. Children affected by ALL are known to have deficits in IQ scores [40] and attention difficulties [40], [41]. Cognitive deficits are typically attributed to chemotherapy with methotrexate which inhibits folate pathway enzymes, and the A1298C variant in *MTHFR* is implied to associate with ADHD risk in 11 cases among 48 ALL patients [42]. Another study also found that the A1298C high-risk allele is more frequent in the 40 Turkish children with ADHD than the 30 controls [43]. However, another larger independent study of 100 Turkish children with ADHD and 300 controls concluded neither C677T nor A1289C alleles contribute to ADHD risk [44].

In our cohort of MM individuals, the C677T and A1298C variants did not associate with the ADHD phenotype, in contrast to the observation reported by Krull et al. [42] and Gokcen et al. [43]. However, these observations are consistent with those found by the family-based IMAGE and PUWMa ADHD genome-wide association (GWA) studies [44], [45]. Several case-control GWA studies (CHOP, NIMH, Utah and IMAGESII) using genotyping arrays (Affymetrix 5.0 and Illumina Hap550K) contain C677T but not A1298C and did not find association between C677T and ADHD. The SNP rs4846049 is not present in the genotyping arrays used by all these ADHD GWA studies therefore it has never been tested for ADHD association. An important difference between the two patient populations is that MM is a neurodevelopmental disorder and cognitive problems in ALL emerge from treatment, so it is an acquired phenotype. The specific mechanism behind the folate reduction is likely distinct. Deficiency in folate among ALL patients is most likely contributed by the presence of the thermolabile MTHFR variants in addition to MTX treatment. Our study indirectly suggests there may be a deficiency in folate in the MM cohort and that it may be secondary to altered levels of the *MTHFR* transcript in addition to the presence of the thermolabile variants 677C>T and/or 1298A>C. Secondly, analysis of linkage disequilibrium of the reference HapMap CEU population shows that the rs4846049 SNP is in linkage disequilibrium (LD; r∧^2^ = 0.931) with the A1298C (rs1801131) (see **Table 6**). It is possible that the association observed in the previous studies was a proxy for the rs4846049 SNP. However, we did not find rs4846049 in LD with A1298C (rs1801131) in Whites with MM (r∧^2^  = 0.634) nor in Hispanics with MM (r∧^2^  = 0.702) in our MM cohort suggesting the present of complex LD correlations among individuals affected by MM.

The rare allele T of rs4846049 is demonstrated to have biological function. A recent study of micro-RNA has shown miR-149 inhibiting the expression of the luciferase reporter engineered with the T allele of the *MTHFR* rs4846049 at the 3′-UTR [46]. In addition, rs4846049 is located approximately 233 bp downstream of a predicted miR-22 target with sequences conserved between human and rodents (chr1:11850598–11850605, TargetScan miRNA Sites, UCSC Genome Browser on Human GRCh37/hg19 Assembly). Exogenous miR-22 has been shown to downregulate expression of *Mthfr* and *Mat1a* in rat liver epithelial cells [47] but the effect on human *MTHFR* is unknown. There are two known SNPs (rs45482794 and rs35737219) located 91bp and 145bp respectively upstream to the miR-22 site in the 3′-UTR of *MTHFR*. Unfortunately, the rare allele frequencies of these two SNPs are too low (∼1 and 3% respectively) to be used in an association study. Further investigations are necessary to verify how individual alleles of rs4846049 affect miR-22 or mir-149 in regulating the expression level of *MTHFR*.

It seems unlikely that elevated ratings on the SNAP-IV, or other tests for ADHD, are simply related to IQ or severity of disease. Burmeister *et al.* showed that there is no difference in level of treatment or treatment revisions, such as hydrocephalus and shunting, between individuals who test positive for ADHD and individuals who do not [7]. In addition, there was no difference in IQ or ambulatory status between those testing positive for ADHD and those testing negative for ADHD [7]. Therefore ADHD status does not appear dependent on IQ, severity of disease and its treatment, or lesion level.

There are several limitations of the current study. One significant limitation is the sample size. However, unlike common diseases such as hypertension, enrolling large samples of MM individuals is relatively difficult because MM is a rare human disorder only affecting 1 in 2,500 live-births in the US. The 478 MM individuals reported here represent the largest sample size to date studied for ADHD who have been genotyped for *MTHFR* gene variants. A second limitation of the study is the confounding factors present in the mixed ethnic backgrounds of the study participants. For example, the rare allele frequencies of C677T and A1298C are significantly different between Whites and Hispanics (see **Table 3**). For this reason, we only performed case-control analyses and present results within each ethnic group. Lastly, we are limited by our knowledge of the LD structure for the *MTHFR* locus. The SNP rs4846049 is a tagSNP selected by the HapMap tagSNP Picker program but it is absent in the majority of the commercial GWA study arrays and was not genotyped for the HapMap Mexican Hispanics. The current report presents results of a pilot study suggesting a possible association of a functional SNP rs4846049 with ADHD among individuals affected by MM. It is necessary to validate the observation in cohorts with large sample sizes and among individuals with and without MM.

In summary, this study demonstrated a positive association between the ADHD phenotype in MM individuals and one SNP, rs4846049, in the 3′-UTR of the *MTHFR* gene. It confirmed results from earlier studies that demonstrate that ADHD behavior is more prevalent in MM individuals than in the general population. These data indicate that *MTHFR* (and, by extension, folic acid) are likely involved in the etiology of ADHD behavior in MM individuals.
